# Ethnic Diversity, Inter-group Attitudes and Countervailing Pathways of Positive and Negative Inter-group Contact: An Analysis Across Workplaces and Neighbourhoods

**DOI:** 10.1007/s11205-017-1570-z

**Published:** 2017-01-28

**Authors:** James Laurence, Katharina Schmid, Miles Hewstone

**Affiliations:** 1 University of Manchester, 2.11 Humanities Bridgeford Street, Oxford Road, Manchester, M13 9PL UK; 2 ESADE Business School, Ramon Llull University, Carrer de Muntaner 318, Pral -1B, 08021 Barcelona, Spain; 3 University of Oxford, South Parks Road, Oxford, OX1 3UD UK

**Keywords:** Ethnic diversity, Prejudice, Positive contact, Negative contact, Communities, Workplaces

## Abstract

This study advances the current literature investigating the relationship between contextual out-group exposure, inter-group attitudes and the role of inter-group contact. Firstly, it introduces the concept of contact-valence into this relationship; that is, whether contact is experienced positively or negatively. Secondly, it presents a comparative analysis of how processes of out-group exposure and frequency of (valenced) contact affect prejudice across both neighbourhoods and workplaces. Applying path analysis modelling to a nationally-representative sample of white British individuals in England, we demonstrate, across both contexts, that increasing out-group exposure is associated with higher rates of both positively- and negatively-valenced contact. This results in exposure exhibiting both positive *and* negative indirect associations with prejudice via more frequent inter-group mixing. These countervailing contact-pathways help explain how out-group exposure is associated with inter-group attitudes. In neighbourhoods, increasing numbers of individuals experiencing positive-contact suppress an otherwise negative effect of neighbourhood diversity (driven partly by increasing numbers of individuals reporting negative contact). Across workplaces the effect differs such that increasing numbers of individuals experiencing negative-contact suppress an otherwise positive effect of workplace diversity (driven largely by increasing numbers of individuals experiencing positive contact).

## Introduction

Concern has long existed that increasing ethnic minority populations may increase inter-ethnic tensions (Blalock 1967). These issues have assumed renewed salience from research suggesting that ethnic diversity may undermine inter group relations and cohesion more widely (Putnam 2007). How societies respond to increasing diversity is (re)emerging as a prominent topic in public/political debates, with potentially far-reaching consequences. For example, anti-immigrant attitudes were believed to significantly influence the United Kingdom’s recent decision to leave the European Union and the success of Donald Trump in the 2016 United States presidential election (Kahn 2016). With immigration and diversity at historically high levels across many developed countries there is a need to focus on the strategies available to address possible emergent tensions.

The contact hypothesis is one way inter-group tensions can be ameliorated, and extensive evidence documents how positive inter-group contact can reduce prejudice (Pettigrew and Tropp 2006; Hewstone 2015). Furthermore, it has been argued that contextual-exposure^1^ to out-groups can reduce prejudice by fostering greater cross-group interaction (Schmid et al. 2014). Micro-level inter-group contact has thus been posited to play a key role in curtailing the drivers of macro-level conflict (Pettigrew 2008). However, in spite of these promising findings, the weight of evidence suggests that increasing out-group populations in an environment tends to result in no difference in, or more negative, out-group attitudes (Dustmann and Preston 2001; Putnam 2007). This has led to criticisms of the efficacy of the contact hypothesis for reducing prejudice in increasingly diverse societies (Forbes 2004; Putnam 2007).

This mixed evidence has triggered significant debate into how higher exposure to out-groups affects prejudice and the role of contact in this relationship. However, two issues potentially confuse this debate. Firstly, studies examining how the size of the ethnic out-group population in an environment affects attitudes have not explored the role of contact-valence in this relationship; that is, how far contact experiences are positive or negative (Hewstone 2015). Instead, studies largely focus on the role of positive, intimate forms of contact, or generic (unvalenced) mixing. However, higher out-group exposure may increase opportunities for both positive *and* negative contact, and while the former can reduce prejudice the latter can increase it (Barlow, et al. 2012). Not accounting for contact-valence may have precluded a proper understanding of the role of contact in the exposure/prejudice relationship.

The second issue is that studies examining the diversity/contact/prejudice relationship have largely focused on residential communities as a context for integration/division. Experiences of ethnic diversity, however, occur across many contexts. One largely overlooked context is the workplace. People in England self-report that their workplaces are more diverse than their neighbourhoods (33% report their workplaces being ‘about half’ or more out-group while 24% report their neighbourhoods are ‘about half’ or more out-group).^2^ Given this degree of diversity, understanding how a greater out-group size in workplaces affects inter-group attitudes is critical.

This study will address these issues. Firstly, we aim to extend the contact hypothesis as applied in contextual-effects studies to consider the role of negatively-valenced contact alongside positively-valenced contact. Secondly, we will examine these processes across both neighbourhoods and workplaces; two main contexts in which individuals spend much of their daily lives. Collectively, this will further our understanding of how out-group exposure affects inter-group relations and the role of inter-group contact in driving/ameliorating positive/negative group relations.

## Theoretical and Analytical Framework

### The Contact Hypothesis and Out-Group Exposure: Theory, Application and Evidence

The ‘contact hypothesis’ broadly stipulates that positive interaction with out-groups undermines prejudice (Allport 1954). Originally, the hypothesis outlined specific conditions under which contact would improve inter-group attitudes, including: equal status, common goal orientated, co-operative contact with the support of authority, law or customs (Allport 1954). Exhaustive individual-level evidence has documented the positive effect of inter-group contact when these conditions are met (although contact not fulfilling these pre-conditions has also been shown to reduce prejudice; Pettigrew and Tropp 2006). Such interaction is partly predicated on opportunities for contact, and positive inter-group contact has been shown to be higher in environments with larger minority shares (Schlueter and Scheepers 2010; Koopmans and Veit 2014; Schmid et al. 2014). Drawing these findings together, research demonstrates that contextual out-group exposure has a positive *indirect* effect on inter-group attitudes via greater inter-group contact (Wagner et al. 2006; Schmid et al. 2014).

**Fig. 1:**
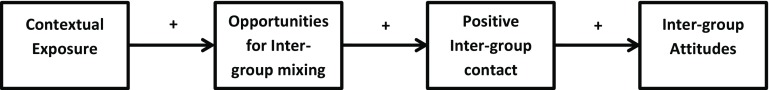
Pathways of the ecological-contact hypothesis

Studies have consequently adapted the individual-level contact hypothesis to derive a contextual-level hypothesis: that individuals living amongst higher proportions of out-groups will report *comparatively more positive* inter-group attitudes than individuals residing amongst lower out-group proportions as a result of greater opportunities for mixing; exposure should therefore have a positive overall^3^ effect on inter-group attitudes (Oliver and Wong 2003; Branton and Jones 2005; Putnam 2007; Bowyer 2008; Legewie 2013; Laurence 2014). Studies frequently label this theory the ‘contact hypothesis’; however, this has led to problems in conflating the individual-level hypothesis with this contextual-level prediction.^4^ To avoid confusion we label the use of the contact hypothesis in this *contextual*-capacity as the ‘ecological-contact hypothesis’, to differentiate it from the individual-level contact hypothesis (Fig. 1).

The ecological-contact hypothesis forms a core part of the theoretical framework used to investigate how out-group size affects prejudice. However, few studies demonstrate evidence supporting its central prediction (*although see* Wagner et al. 2006). Instead, studies tend to demonstrate no association (Stein et al. 2000; Laurence 2014) or a negative relationship between contextual-exposure and inter-group attitudes (Taylor 1998; Dustmann and Preston 2001; Putnam 2007; Schneider 2008; Ayers et al. 2009; Schlueter and Scheepers 2010). Negative effects of increasing ethnic diversity have also been demonstrated longitudinally (Laurence and Bentley 2016). These findings are largely explained with reference to the threat hypothesis; that is, an increasing minority-share in an environment generates hostility amongst the majority group, due to actual/perceived resource competition (Blalock 1967).

The extant literature thus shows evidence of positive *indirect* effects of contextual-exposure on inter-group attitudes via contact but also negative *overall* effects of exposure. More recently, attempts have been made to reconcile these potentially contradictory findings by suggesting that exposure exerts both positive *and* negative effects on inter-ethnic attitudes via competing mechanisms of contact *and* threat (Schlueter and Scheepers 2010; Pettigrew 2013; Laurence 2014). Studies provide support for this idea, simultaneously demonstrating that while higher exposure has a positive indirect association with inter-group attitudes via contact it also exhibits direct negative associations with out-group trust and inter-group attitudes, as well as positive associations with perceived-threat (Schlueter and Wagner 2008; Schmid et al. 2008, 2014; Pettigrew et al. 2010; Schlueter and Scheepers 2010). Further decomposing the overall relationship between ethnic diversity and prejudice, studies show how out-group size can often appear to have no overall effect on inter-group attitudes. However, once inter-group contact is adjusted for, exposure exhibits a significant, negative *direct* effect on inter-group attitudes (Stein et al. 2000; Schneider 2008; Semyonov and Glikman 2009; Legewie 2013; Laurence 2014). In other words, the greater contact reported in more diverse environments suppresses^5^ an otherwise negative effect of exposure on inter-group relations, largely attributed to processes of perceived threat.

In sum, two key conclusions are drawn from this current research. Firstly there is evidence that exposure exhibits *both* positive *and* negative associations with indicators of inter-group attitudes, emerging from two competing pathways: a positive, behavioural pathway via inter-group contact and a negative, psychological pathway via perceived-threat. Secondly, that the more frequent inter-group contact experienced at higher exposure appears to counteract an otherwise negative impact of exposure on attitudes, attributed to a pathway of perceived-threat. We argue that this picture may be incomplete.

One problem with prior research concerns the adaptation of the individual-level contact hypothesis to the contextual-level. In particular, the core prediction, at the centre of the ecological-contact hypothesis, that greater inter-group contact with exposure will *only* drive positive outcomes. This fails to account for contact *quality*, and the possibility that negative inter-group contact may increase prejudice.

### Out-Group Exposure and Contact Quality

Contact ‘*quality* reflects the extent to which face-to-face intergroup encounters are experienced positively or negatively’, where experiences of the former decrease prejudice while experiences of the latter increase prejudice (Lolliot et al. 2015: 654). The extent to which contact experiences are positive/negative is conceived of as the ‘valence’ of contact. As noted above, from its inception, the quality of inter-group contact for attitude-change formed a foundation of the contact hypothesis: contact alone was not believed to reduce prejudice but only contact under ‘optimal’ conditions; at the same time, sub-optimal contact could increase prejudice (Allport 1954; Amir 1969).

Numerous factors may affect whether contact is experienced positively/negatively. Out-group contact may be positive or negative depending on whether it involves, for example, being harassed, intimidated, or insulted (Aberson and Gaffney 2009), being welcomed (Lolliot et al. 2015), or being helped/pestered (Stephan et al. 2002; Pettigrew 2008). Relatedly, contact-valence can be determined by the ‘situational contexts’ of the encounter, with non-superficial, equal status, and voluntary contact shown to be related to more positive encounters (and their inverse to negative encounters) (Allport 1954; Pettigrew 2008: 192). Individuals may also have predispositions to experience any out-group encounters they do have more positively/negatively. For example, individuals with higher levels of authoritarian personality traits, greater out-group anxiety and perceived threat appear more likely to experience negative out-group encounters (Stephan and Stephan 1985; Pettigrew 2008).

At the *individual*-level, studies demonstrate how inter-group mixing can be experienced both positively and negatively, and that while the former reduces prejudice the latter can increase it (Islam and Hewstone 1993; Pettigrew 1998; Paolini et al. 2010; Barlow et al. 2012). However, discussion or measurement of contact-quality has been almost entirely absent from the literature exploring the role of contact in the contextual out-group exposure/prejudice relationship (*although see* Koopmans and Veit (2014) and Koopmans and Schaeffer (2015) who examined local cohesion outcomes). Instead, studies into the contextual-effects of diversity either test the role of positive, intimate measures of contact (e.g. presence of/interaction with out-group friends; Wagner et al. 2006; Schlueter and Scheepers 2010; Laurence 2014), or apply generic (unvalenced) measures of contact (e.g. frequency of mixing with out-groups; Stein et al. 2000; Schmid et al. 2014). The difficulty here is that those studies assessing positive contact do not account for negative contact, while studies assessing generic measures of contact (implicitly) treat these as positive contact, potentially conflating positive and negative experiences.

**Fig. 2:**
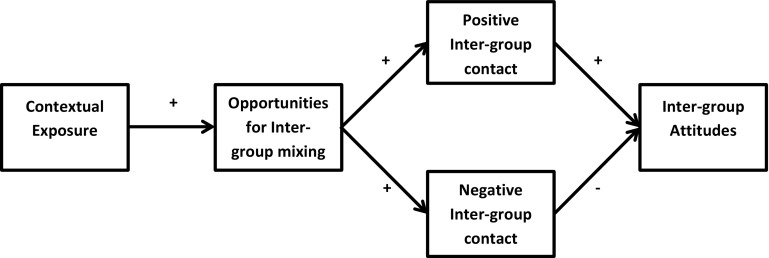
Countervailing pathways of the adapted ecological-contact hypothesis

This potential weakness in the current literature lies at the core of the ecological-contact hypothesis, which assumes that higher contact with increasing out-group exposure will only generate positive effects on attitudes. Yet, studies demonstrate that exposure can increase the frequency of both positive and negative contact experiences (Pettigrew 2008; Koopmans and Veit 2014). These dual processes of positive/negative encounters can also be observed at the macro-level, where rates of inter-ethnic marriage but also inter-ethnic crime increase with greater inter-group spatial propinquity (Blau and Schwartz 1984; South and Messner 1986). Drawing on these studies, we thus aim to modify the current ecological-contact hypothesis and integrate this (currently absent) negative-contact pathway into the contextual-exposure/inter-ethnic attitudes relationship. To wit, exposure increases opportunities for inter-group mixing; however, this may increase the frequency of *both* positive *and* negative contact. While the former should improve inter-group attitudes, the latter likely harms them. This will result in *dual countervailing indirect contact*-*pathways*, which operate to both reduce but also increase prejudice (*see* Fig. 2).

In sum, studies have examined: how contextual-exposure affects prejudice via positive and/or generic inter-group contact pathways (e.g. Wagner et al. 2006); how positive and negative contact *at the individual*-*level* is associated with prejudice (e.g. Paolini et al. 2010; Barlow et al. 2012); and how contextual exposure predicts frequency of positive *and* negative contact (Pettigrew 2008). Yet, no study has brought these elements together to look at the role of positive *and* negative contact for understanding how exposure affects inter-ethnic attitudes. This precludes a fuller understanding of how exposure affects prejudice, and the role of contact in this process.

### Workplace Exposure and Inter-Group Attitudes

A second issue with the current exposure/contact/prejudice literature is the predominant focus on residential communities to test how contextual-exposure to out-groups affects inter-group relations (although there is significant work on school-exposure/prejudice, e.g. Dejaeghere et al. 2012). Processes of contextual-exposure and contact may not operate equally across all contexts. Furthermore, selection into/out of non-neighbourhood contexts, based on ethnic composition, may be less active, providing potentially more robust estimates of the effects of exposure. One context receiving insufficient attention is the workplace.

On one hand, higher workplace ethnic diversity may be more efficacious for cultivating positive attitudes than out-group exposure across neighbourhoods. Increasingly diverse workplaces (compared to neighbourhoods) may lead to more inter-group mixing as team composition, task assignment, and the necessity of interaction with co-workers may restrict tendencies towards homophily (Kokkonen et al. 2014). Workplace interaction could also fulfil more of Allport’s (1954) conditions for positive contact, e.g. co-operative interaction, working towards common goals (Estlund 2003, 2005; Kokkonen et al. 2014). The necessity of repeated interactions with colleagues, informal norms of behaviour in work places, alongside formalised codes of conduct (e.g. anti-bullying codes and diversity policies), may also limit the potential for negative contact experiences (Estlund 2003). As such, increasing workplace diversity could lead to more, and more positive, inter-group mixing, undermining prejudice.

On the other hand, increasing workplace diversity could lead to more negative inter-group attitudes compared to neighbourhood diversity. Contact in workplaces may be more casual/superficial (Amir 1969). It could be more involuntary, competitive, or involve larger status differentials (e.g. organisational hierarchical stratification aligned with ethnicity; Allport 1954; Amir 1969; Islam and Hewstone 1993). Furthermore, because the workplace restricts tendencies towards homophily, individuals predisposed to experience contact negatively may be compelled into negative contact where other contexts would allow them to avoid it. As such, workplace exposure may generate comparatively more negative outcomes.

Few studies have examined how workplace out-group size affects contact and prejudice. As with residential communities, studies have shown that self-reported workplace diversity is associated with positive forms of out-group contact (e.g. friendship ties; Ibarra 1995; Kokkonen et al. 2014). They have also found that intimate contact with workplace colleagues is associated with greater tolerance (Frølund Thomsen 2012). Accordingly, Wagner et al. (2006) show that higher workplace diversity has an indirect positive association with inter-group attitudes: higher workplace diversity is associated with greater personal contact with out-groups in the workplace, which is associated with more (intimate) contact with out-group friends, which predicts lower prejudice.

In spite of such positive *indirect* associations however, few studies have tested whether individuals in more diverse workplaces report more or less prejudice than those in more homogeneous workplaces (or individuals not in employment); that is, what is the *overall* effect of workplace exposure on attitudes. Large survey studies report mixed findings. While Escandell and Ceobanu (2008) find that perceived workplace diversity in Spain is *not* associated with attitudes towards immigrants, a cross-national comparative study found a positive effect of perceived workplace diversity on reduced anti-immigrant feelings (Strabac 2011). Moreover, ethnographic work highlights the efficacy of working together for building inter-group relations, compared to more superficial neighbourhood contact (Newman 1999; Matejskova and Leitner 2011). However, there is a dearth of analysis examining *how* workplace exposure affects inter-group relations via positive and/or negative contact experiences, and how these effects may differ from neighbourhood contexts.

### Hypotheses

Based on the current literature, we will generate hypotheses under three key aims. The first aim is to explore the role of contact-valence in the contextual-diversity/prejudice relationship. Firstly, we posit that increasing out-group exposure will be associated with more positive *and* with more negative inter-group contact (H1). Secondly, positive inter-group contact will be positively associated with inter-group attitudes, while negative inter-group contact will be negatively associated with them (H2). Based on H1 and H2, out-group exposure will exert a positive *indirect* effect on inter-group attitudes via positive contact, but also a negative *indirect* effect via negative inter-group contact (H3).

The second aim is to test how these contact pathways help us to understand the *overall*-effects of contextual diversity on prejudice. Previous work demonstrates that the indirect positive effect of out-group size on inter-group attitudes via inter-group contact suppresses an otherwise direct negative effect of exposure on attitudes, which is nominally attributed to perceived-threat. However, drawing on our predictions above, we expect that only after controlling for positive inter-group contact in particular (and not all contact) will a negative effect of contextual-exposure emerge (H4a); and that any emergent negative effect of out-group exposure on attitudes will (in part) be accounted for by the frequency of negative contact (H4b).

The third aim is to explore how far these processes operate differently across different sites. We suggest that out-group exposure may exhibit different associations with inter-group contact and inter-group attitudes across workplaces and neighbourhoods: theoretically, workplace diversity could lead to comparatively more positive or more negative inter-group relations compared to neighbourhoods. However, as outlined, there is little prior research that directly compares workplace and neighbourhood effects; thus, we do not hypothesise as to how these effects will differ, taking a more exploratory approach.

## Data and Methods

### Sample

This study uses the 2010 Managing Cultural Diversity Survey (MCDS) of 16+ year olds in England. This is a two-stage, random-location, nationally-representative sample, conducted using face-to-face interviews. Middle Super Output Areas (MSOAs) (which contain around 7000 residents) formed the Primary Sampling Units (PSUs) and then a random sample of residents, 16 or older, was selected within each MSOA.^6^ Any (non-self-reported) contextual-level data is taken from the 2011 UK Census. The current study focuses on the white British sample, since the theoretical framework (especially the threat hypothesis) has greater applicability to higher-status groups (Oliver and Wong 2003); processes of inter-group contact do, however, operate for minority as well as majority groups (Pettigrew and Tropp 2006; Barlow et al. 2013).

### Key Variables

#### Out-Group Attitudes

To measure out-group attitudes we use a ‘feeling thermometer’ measure. Individuals were presented with a show card of a thermometer running from 0 to 100. On the thermometer, 0 was labelled as ‘cold’, 50 was labelled as ‘neutral’, and 100 was labelled as ‘warm’. Respondents were then asked: ‘Please rate how YOU feel about the following groups on a thermometer that runs from zero to a hundred degrees. The higher the number, the warmer or more favourable you feel. The lower the number, the colder or less favourable you feel. How do you feel about people from an ethnic minority background?’

#### Workplace and Neighbourhood Out-Group Exposure

It is not possible to access data on UK workplace diversity to link to respondents. As such, as applied in previous studies in the literature, we use self-reported workplace exposure measures. For comparability between contexts we also apply self-reported neighbourhood measures. For the workplace (asked of those in employment) this is: ‘What proportion of people in your workplace are from an ethnic minority background?’ For the neighbourhood this is: ‘What proportion of people with an ethnic minority background live in your neighbourhood?’ 5-point responses range from 1 (*none*) to 5 (*almost all or all*). However, we can also measure the statistical out-group size of our respondents’ community (at the Middle Super Output Area-level) using UK census data, and explore whether substantive differences emerge between perceived-/actual-neighbourhood diversity.^7^

#### Positive and Negative Contact

Contact-valence has been conceptualised in two ways. The first approach separates positive and negative ‘specific incidents’ that occur in an individual’s daily life by asking respondents to report the frequency of positive incidents (e.g. being helped by out-groups) and negative incidents (e.g. being pestered by out-groups) experienced (Pettigrew 2008). The second way defines contact in terms of ‘overall-valence’. To measure this surveys ask about the ‘quantity of contact’ an individual has, and then how ‘positive or negative’ or ‘enjoyable or not enjoyable’ this contact was (Barlow et al. 2012). Under the ‘specific-incident’ approach, an individual can experience *both* positive and negative contact. Under the ‘overall-valence’ approach, individuals only experience uni-valenced contact (i.e. positive *or* negative); however, different individuals can experience contact positively or negatively.

Both modes of conceptualisation have relative advantages and likely pick up overlapping but also distinct processes. However, the logic behind the ‘overall-valence’ approach is that positive/negative contact experiences are ‘multifaceted phenomena’ and cannot solely be defined by specific incidents (e.g. being pestered; Barlow et al. 2012). Instead, all out-group encounters can engender positive/negative emotions for individuals and it is these collective experiences with out-groups which generate an “overall perceived valence of [out-group] interactions” which distinguishes whether one’s contact is positive or negative (Barlow et al. 2012, p1631). Our analysis of the MCDS data takes this ‘overall-valence’ approach to conceptualising positive/negative contact, given the available measures in this data.

One key advantage of the MCDS data is our ability to measure not only the degree of out-group exposure in a specific context (neighbourhood/workplace) but also both the amount of contact an individual experiences *in each context* as well as the valence of this contact. We therefore have two sets of context-specific measures capturing contact quantity and quality (one set for the neighbourhood and one set for the workplace). Individuals are first asked the quantity of out-group contact they have at each site. Generic workplace contact-quantity is measured by: ‘How often, if at all, do you mix with people from *an ethnic minority background* in your workplace?’ This was asked of those in work reporting that at least ‘a few’ people in their workplace are from an ethnic minority background. Generic neighbourhood contact-quantity is measured by: ‘How often, if at all, do you mix socially with people from *an ethnic minority background* in your neighbourhood?’ This was asked of all people.^8^ Responses range from 1 (*Never*) to 5 (Very often) on a 5-point scale.

Following the question on contact quantity, individuals rated the context-specific ‘enjoyableness’ of this contact. For the workplace the measure is: ‘How much, if at all, do you enjoy this mixing with people from *an ethnic minority background* in your workplace?’ To test neighbourhood contact-valence individuals were asked: ‘How do you feel about mixing socially with people from *an ethnic minority background* within your neighbourhood?’ Responses to both questions include: ‘I enjoy it a great deal’, ‘I enjoy it quite a bit’, ‘I enjoy it a little’, ‘I don’t enjoy it very much’, and ‘I don’t enjoy it at all’. These questions were only asked of those who reported mixing ‘very rarely’ or more.

As outlined by the ‘overall-valence’ approach to positive/negative contact, we thus find that for some individuals the mixing they experience can be ‘enjoyable’ (positive), while for other individuals the mixing they experience can be ‘not enjoyable’ (negative). However, previous studies demonstrate that what matters in particular is the *amount* of positive/negative contact; not just if an individual has positive/negative contact (Barlow et al. 2012). Accounting for this is important because not only will the number of individuals who report having positive/negative contact likely increase with more diversity, but the frequency of contact these individuals experience should also increase given greater opportunities for mixing.

We therefore need to operationalise these measures to allow us to capture the effects of *frequency* of positive/negative contact amongst the sample. One option is to generate an interaction-term between contact-quantity and -quality to test how frequency of differently-valenced contact matters for prejudice. However, this would exclude respondents who reported ‘no contact,’ because they provide no data on the contact-quality measure^9^. Instead, we combined the questions on both contact-quantity and -quality to generate separate measures of the frequency of valenced-contact.^10^ These draw together *whether* an individual experiences contact, *if* the contact they experience is more positive *or* negative, and, importantly, *how much* of it they experience. We outline our process below.

Using the measure of ‘enjoyableness’ of contact, we first created three separate binary variables which divide up individuals by whether they experienced: ‘low enjoyment’ contact (if an individual reported ‘don’t enjoy it very much’ or ‘don’t enjoy it at all’); ‘medium enjoyment’ contact (if an individual reported ‘enjoy it a little’ or ‘enjoy it quite a bit’); and ‘high enjoyment’ contact (if an individual reported ‘enjoy it a great deal’).^11^ For each of these binary variables, those who did experience that valence of contact are coded as 1; if an individual did not experience this type of contact they are coded as 0. Next, for individuals who are coded as ‘1’ in each variable, we replaced this value of ‘1’ with that same individual’s actual contact-quantity value (1–5). Thus, we obtain three separate variables measuring the quantity of contact reported by individuals who reported the valence as either high, medium or low-enjoyment, respectively. Individuals who reported no contact are then coded as 0 in each separate measure.

Taking the ‘low-enjoyment’ contact variable as an example, this variable is coded 0–5. Values of 1–5 reflect the *quantity* of contact experienced by those individuals who reported ‘low-enjoyment’ contact; essentially, it is the contact-quantity variable but restricted to those individuals who reported their contact experiences to be ‘low-enjoyment’. The 0 category of this variable represents individuals who ‘never’ experienced ‘low-enjoyment’ contact: that includes, firstly, those individuals who reported ‘no contact at all’, and secondly, those individuals who did have contact but who reported that it was either ‘medium-’ or ‘high-enjoyment’ contact. This yields a measure of the frequency of low-enjoyment contact in the sample (with those individuals who reported no contact, ‘medium-enjoyment’ contact or ‘high-enjoyment’ contact coded as ‘never’ having ‘low-enjoyment’ contact). This process was repeated for the ‘medium-’ and ‘high-enjoyment’ measures, capturing the contact-quantity for those who reported their contact was either ‘medium-’ or ‘high-enjoyment’.

As noted, under the ‘overall-valence’ approach to measuring positive/negative contact, an individual only experiences one form of valenced contact. However, in spite of this, all individuals are present in (i.e. are coded into) each variable: if they have contact ‘very rarely’ or more in one variable then they will have a 0 value in the other variables, alongside those who had ‘no contact at all’ who are coded as 0 *in all three variables*.

This produces a set of three new variables, one for the neighbourhood and one for the workplace, respectively;

1. The amount of (context-specific) mixing experienced by those individuals who reported that their contact was ‘high-enjoyment’;
2. The amount of (context-specific) mixing experienced by those individuals who reported that their contact was ‘medium-enjoyment’’
3. The amount of (context-specific) mixing experienced by those individuals who reported that their contact was ‘low-enjoyment.’

### Controls

At the individual-level we include: age; children under 18 in household; number of people in the household; marital status; employment status; gender; qualifications; number of years in area; housing tenure; and social grade of the main household earner. In our neighbourhood analysis we also control for characteristics of individuals’ communities (MSOA). Using factor analysis we generate three indices of: status disadvantage (reverse coded percent in managerial/professional occupations and percent with degrees: *Eigen Value* 1.89); resource disadvantage (percent of households social renting, percent of households with female lone-parent, percent of economically active unemployed: *Eigen Value* 1.97); and urbanisation (percent aged 65+, density (persons per hectare) and turnover (rate of inflow plus outflow per 1000 people between mid-2009 and mid-2010): *Eigen Value* 1.80). Unfortunately, we have no data on the characteristics of respondents’ workplace, e.g. education amongst co-workers.

### Analytic Approach

This paper has three key aims. The first aim is to explore our central contention that increasing ethnic diversity will exert both positive *and* negative indirect effects on inter-group attitudes via pathways of contact. As outlined, this paper takes an ‘overall-valence’ approach to measuring positive/negative contact, in which individuals only experience one form of valenced-contact, but different individuals can experience different forms of valenced-contact. Applying this conception to our central contention, we predict that: increasing exposure will lead *some* individuals to experience more frequent positive contact, and these individuals will report more out-group warmth (a positive indirect-effect); however, it will also lead *other* individuals to experience more frequent negative contact, and these individuals will report less out-group warmth (a negative indirect-effect). To formally test this we take a structural equation modelling approach, applying path analysis models as used in the literature (Wagner et al. 2006; Schlueter and Wagner 2008; Schmid et al. 2014). We use the bootstrap method to estimate the indirect effects with bias-corrected confidence intervals, based on 5000 bootstrap samples, which will allow us to test multiple mediators simultaneously^12^ (Preacher and Hayes 2008).

The second key aim is to explore the role of (valenced) contact in understanding the overall association between diversity and inter-group attitudes. To examine this question we will run a baseline model testing the relationship between diversity and attitudes, and then enter our measures of (valenced) contact into the model in a stepwise fashion. Thus, we can explore how the (strength and significance of the) relationship between contextual-diversity and attitudes changes after accounting for rates of positive/negative contact. This will allow us to test how far differently-valenced contact may be driving/suppressing any positive/negative direct-effects of diversity.

The third aim is to explore whether measures of diversity, contact and prejudice have different associations across workplaces and neighbourhoods. We will therefore repeat all analyses separately for neighbourhoods and workplaces.^13^

To best examine the impact of accounting for contact-valence vis-à-vis the use of generic measures of contact, we will conduct all analyses in two stages. Firstly, we will undertake a replication of the dominant approach in the current literature: testing the mediating role of frequency of (generic) contact in the diversity/prejudice relationship. Secondly, we will replace this generic measure with our measures of valenced-contact, which disaggregates generic-contact by its level of enjoyableness. This will provide opportunities to observe how accounting for contact-valence may alter our understanding of the effects of diversity via contact, relative to the current literature.

For the neighbourhood analysis we analyse the impact of exposure to out-groups in the neighbourhood amongst all White British respondents. For workplaces, we examine how being in a more diverse workplace is related to prejudice compared to individuals in less diverse workplaces as well as individuals not in a workplace. Therefore, individuals not in work are coded as having ‘no workplace exposure’, alongside individuals in homogeneous workplaces. As our models control for employment status, alongside other socio-demographics, this should adjust for differences between these in/out of work ‘no exposure’ individuals.^14^

We apply linear regression models with clustered standard errors at the MSOA-level. Our workplace models only include individual-level variables. Our neighbourhood models also contain level-2 variables (e.g. MSOA-disadvantage). For comparability across models we report results from the linear regression/clustered standard error models. However, we replicate models using a Hierarchical Linear Modelling (HLM) framework for robustness.

## Results

### Neighbourhood Diversity

We begin by analysing these relationships across neighbourhoods (Table 1). Model 1 generates our baseline model, testing the overall-association between neighbourhood exposure and inter-group attitudes. We observe that neighbourhood diversity exhibits a negative but non-significant association with out-group warmth. Drawing on the approach of the current literature, we next explore the mediating role of *generic* contact in this relationship. We observe that at higher diversity individuals report more frequent generic inter-group contact (Model 2), while individuals who report more frequent generic contact report warmer out-group attitudes (Model 3). Testing these pathways simultaneously using a structural equation modelling approach (Model 4), we find a positive indirect-effect of diversity on out-group warmth driven by those individuals reporting more frequent generic contact:^15^ *b* = 0.972 (95% bias-corrected CIs = 0.53, 1.55). Therefore, despite the non-significant negative overall-association between exposure and inter-group attitudes (*see* Model 1) exposure does have a positive indirect-effect via individuals who report greater neighbourhood mixing. Prior studies find that such positive indirect-effects via contact can suppress an otherwise direct negative association between exposure and attitudes. If we compare the neighbourhood diversity coefficient between Model 1 and Model 3, when we adjust for individuals’ higher out-group mixing in diverse neighbourhoods the negative diversity coefficient nearly doubles and becomes significant.

**Table 1:** Neighbourhood diversity, neighbourhood (generic) inter-group contact and out-group attitudes

Model type	Model 1	Model 2	Model 3	Model 4	
	OLS	OLS	OLS	SEM	
Outcome	Feeling thermometer	Generic neighbour mixing	Feeling thermometer	Generic neighbour mixing	Feeling thermometer
SES disadvantage	−1.682	0.104	−2.004	0.101	−1.999
	(1.509)	(0.079)	(1.482)	(0.071)	(1.338)
Urbanisation	3.270**	0.061	3.081**	0.054	3.093**
	(1.128)	(0.073)	(1.118)	(0.059)	(1.101)
Skill disadvantage	1.543	−0.067	1.750	−0.057	1.725
	(1.368)	(0.075)	(1.352)	(0.070)	(1.309)
Neighbourhood diversity	−1.073	0.300***	−2.001*	0.316***	−2.020*
	(0.946)	(0.057)	(0.954)	(0.048)	(0.932)
Generic neighbour mixing			3.093***		3.073***
			(0.631)		(0.675)
Constant	45.572***	1.723***	40.245***	1.917***	39.876***
	(8.453)	(0.468)	(8.659)	(0.405)	(7.727)
N	773	773	773	773	773

Significance levels: ^+^ < 0.1; * 0.05; ** 0.01; *** 0.001; models contain all individual-level co-variates (although not shown); clustered standard errors; unstandardized coefficients

These findings lead us to draw similar conclusions to prior studies: neighbourhood exposure has a positive indirect-effect on attitudes via frequent contact at higher exposure; and this positive indirect-effect suppresses an otherwise negative effect of exposure, which is independent of inter-group contact. However, this conclusion assumes all mixing is positive (as one might infer given the positive coefficient of generic mixing). We next explore how accounting for contact-valence affects our understanding of this contextual-exposure/contact/prejudice relationship by substituting the measure of generic contact for our measures of low-, medium- and high-enjoyment contact (Table 2).

**Table 2:** Neighbourhood diversity, neighbourhood (valenced) inter-group contact and out-group attitudes

Model type	Model 1	Model 2	Model 3	Model 4	Model 5
	OLS	OLS	OLS	OLS	OLS
Outcome	Feeling therm.	‘High enjoy’ neighbour mixing	‘Medium enjoy’ neighbour mixing	‘Low enjoy’ neighbour mixing	Feeling therm.
SES Disadvantage	−1.682	−0.010	0.086	0.009	−1.720
	(1.509)	(0.069)	(0.080)	(0.015)	(1.464)
Urbanisation	3.270**	0.043	0.062	−0.015	2.784*
	(1.128)	(0.061)	(0.066)	(0.016)	(1.097)
Skill disadvantage	1.543	0.036	−0.086	−0.009	1.484
	(1.368)	(0.070)	(0.077)	(0.013)	(1.340)
Neighbourhood diversity	−1.073	0.117*	0.140*	0.035*	−1.422
	(0.946)	(0.052)	(0.058)	(0.017)	(0.926)
‘High enjoy.’ Neighbour mixing					3.912***
					(0.802)
‘Medium enjoy.’ Neighbour mixing					2.209**
					(0.696)
‘Low enjoy.’ Neighbour mixing					−12.069***
					(2.923)
Constant	45.572***	−0.342	0.822^+^	0.074	45.989***
	(8.453)	(0.449)	(0.489)	(0.087)	(8.531)
N	773	773	773	773	773

Significance levels: ^+^ < 0.1; * 0.05; ** 0.01; *** 0.001; models contain all individual-level co-variates (although not shown); clustered standard errors; unstandardized coefficients

We observe again the non-significant, negative association between diversity and out-group warmth (Model 1, Table 2). Models 2–4 then test the association between neighbourhood diversity and frequency of high-, medium- or low-enjoyment contact. We find a positive relationship between diversity and the number of individuals who report more frequent positive forms of contact (i.e. high and medium enjoyment). However, we also observe that the number of individuals reporting more frequent negative forms of contact (i.e. low enjoyment) also increases with diversity. Therefore, behind the positive association between diversity and generic-contact (Model 2, Table 1) we find that, in more diverse neighbourhoods, some individuals report more frequent positive contact, but other individuals report more frequent negative contact (although exposure is more strongly associated with positive contact in our sample).^16^ A large part of the positive association between diversity and generic-contact is thus driven by individuals who report more frequent ‘high-’ and ‘medium-enjoyment’ contact; nonetheless, part of the association is driven by other individuals reporting more frequent ‘low-enjoyment’ contact.^17^ These results provide support for Hypothesis 1 (H1).

We next examine the association between measures of ‘low-’, ‘medium-’ and ‘high-enjoyment contact’ and out-group warmth (Model 5). Individuals reporting more ‘high-enjoyment’ contact exhibit more positive attitudes; individuals reporting more ‘medium-enjoyment’ contact also exhibit more positive attitudes (the association is weaker, yet not significantly so); however, individuals reporting more ‘low-enjoyment’ contact exhibit more negative attitudes. These results provide support for H2.

At higher exposure both positive and negative contact are more frequent; however, while the former is positively associated with attitudes the latter is negatively so. We posited that these pathways would result in diversity exerting both positive and negative indirect-effects on attitudes via *contact*. To explore these effects we model the pathways simultaneously in a structural equation model.

**Fig. 3:**
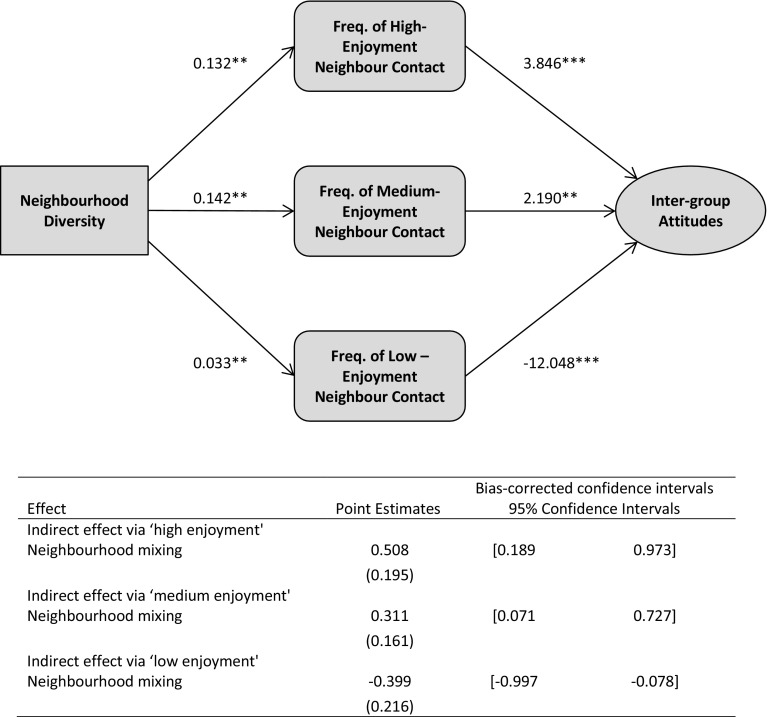
Mediation model of the effect of neighbourhood diversity on out-group attitudes through frequency of ‘low-’, ‘medium-’ and ‘high-enjoyment’ inter-group contact. *Notes* unstandardized coefficients are shown, and bootstrap standard errors are given in parentheses; when confidence intervals do not include zero, value of the indirect effect is significant at the *p* ≤ 0.05 level; all results were controlled for individual-level and community-level covariates

Figure 3 summarises this model, showing the unstandardized coefficients for each pathway and the indirect-effects. Neighbourhood diversity has positive indirect-effects on out-group warmth via those individuals who report more frequent ‘medium-’ and ‘high-enjoyment’ mixing at higher exposure (although the effects are not significantly different). However, diversity also has a negative indirect-effect on out-group warmth via those individuals who report more frequent ‘low-enjoyment’ contact at higher exposure.^18^ This confirms our predictions in H3.

We next explored what role these countervailing indirect-effects play for understanding the overall effect of neighbourhood diversity on prejudice. To do so we added the valenced-contact measures into our models in a stepwise fashion to observe the relative change in the size/significance of the exposure coefficient (Table 3). As previously observed, neighbourhood exposure has a negative but non-significant overall association with inter-group attitudes (Model 1, Table 3). We first include frequency of ‘high-enjoyment’ mixing in the model (Model 2): compared to Model 1 the negative effect of neighbourhood exposure increases by 35%. Next we included frequency of ‘medium-enjoyment’ contact: the negative effect of neighbourhood exposure increases by a further 40% and is now significant (Model 3).

**Table 3:** Changing effects of neighbourhood diversity with (valenced) inter-group contact

Model type	Model 1	Model 2	Model 3	Model 4
	OLS	OLS	OLS	OLS
Dependent variable	Feeling therm.	Feeling therm.	Feeling therm.	Feeling therm.
SES disadvantage	−1.682	−1.650	−1.887	−1.720
	(1.509)	(1.497)	(1.476)	(1.464)
Urbanisation	3.270**	3.135**	2.896*	2.784*
	(1.128)	(1.124)	(1.118)	(1.097)
Skill disadvantage	1.543	1.428	1.628	1.484
	(1.368)	(1.364)	(1.356)	(1.340)
Neighbourhood diversity	−1.073	−1.439	−2.006*	−1.422
	(0.946)	(0.959)	(0.943)	(0.926)
‘High enjoy.’ Neighbour mixing		3.140***	4.503***	3.912***
		(0.733)	(0.819)	(0.802)
‘Medium enjoy.’ Neighbour mixing			2.909***	2.209**
			(0.697)	(0.696)
‘Low enjoy.’ Neighbour mixing				−12.069***
				(2.923)
Constant	45.572***	46.647***	44.724***	45.989***
	(8.453)	(8.589)	(8.655)	(8.531)
N	773	773	773	773

Significance levels: ^+^ < 0.1; * 0.05; ** 0.01; *** 0.001; models contain all individual-level co-variates (although not shown); clustered standard errors; unstandardized coefficients

Thus, after accounting for the positive indirect-effects of exposure via those individuals who report more frequent ‘high-’ and ‘medium-enjoyment’ mixing, neighbourhood diversity has a significant direct negative association with inter-group attitudes. One possibility is that this negative association is being driven (in part) by those individuals who report more ‘low-enjoyment’ mixing at higher exposure. On adding ‘low-enjoyment’ mixing into our model (Model 4), the negative effect of neighbourhood exposure is reduced by 30% and rendered non-significant. These findings support our predictions in H4a and H4b.

### Workplaces

Our next aim is to explore how these processes operate across workplaces (Table 4). Model 1 creates our baseline model for workplaces, testing the overall association between workplace diversity and out-group warmth. We observe that, unlike neighbourhoods, workplace exposure has a positive (but non-significant) overall association with inter-group attitudes.^19^ The next step is to explore the mediating role of *generic* workplace contact in this relationship. Model 2 shows a strong association between workplace diversity and frequency of (generic) workplace mixing (over twice as strong as the association found in neighbourhoods^20^), and Model 3 shows generic workplace mixing is positively associated with attitudes.^21^ Testing these pathways simultaneously in a structural equation model (Model 4) reveals a positive indirect-effect of diversity on out-group warmth via generic contact: *b* = 1.624 (95% bias-corrected CIs = 0.102, 3.41). Therefore, as across neighbourhoods, there is a positive *indirect*-effect of workplace diversity via mixing. However, unlike neighbourhoods, this positive indirect-effect is not suppressing an otherwise negative direct impact of diversity on attitudes: comparing the workplace diversity coefficient between Model 1 and Model 3, we find that once generic workplace mixing is accounted for, the (non-significant) positive coefficient for workplace exposure is reduced to near zero.

**Table 4:** Workplace diversity, workplace (generic) inter-group contact and out-group attitudes

	Model 1	Model 2	Model 3	Model 4	
	OLS	OLS	OLS	SEM (OLS)	
	Feeling therm.	Generic work mixing	Feeling therm.	Generic work mixing	Feeling therm.
Workplace diversity	1.981	0.796***	0.357	0.795***	0.357
	(1.302)	(0.045)	(1.596)	(0.042)	(1.480)
Generic work mixing			2.041^+^		2.048^+^
			(1.226)		(1.137)
Constant	38.808***	1.187***	36.385**	1.187***	36.385***
	(11.028)	(0.241)	(11.108)	(0.223)	(10.302)
N	773	773	773	773	773

Significance levels: ^+^ < 0.1; * 0.05; ** 0.01; *** 0.001; models contain all individual-level co-variates (although not shown); clustered standard errors; unstandardized coefficients; *, **, ** used within the tables themselves to signify different levels of statistical significance of the coefficients

Drawing on these findings, we might conclude that workplace exposure has a positive indirect-effect on attitudes via mixing and that this drives almost all of the positive (albeit non-significant) overall association between exposure and attitudes. However, again, these conclusions assume all mixing is positive. We next explore the role that differently valenced-contact plays across workplaces (Table 5).

**Table 5:** Workplace diversity, workplace (valenced) inter-group contact and out-group attitudes

Model type	Model 1	Model 2	Model 3	Model 4	Model 5
	OLS	OLS	OLS	OLS	OLS
Outcome	Feeling therm.	‘High enjoy’ work mixing	‘Medium enjoy’ work mixing	‘Low enjoy’ work mixing	Feeling therm.
Workplace diversity	1.445	0.428***	0.592***	0.091*	1.072
	(1.226)	(0.080)	(0.077)	(0.042)	(1.451)
‘High enjoy’ work mixing					3.013**
					(1.023)
‘Medium enjoy’ work mixing					−0.484
					(1.045)
‘Low enjoy’ work mixing					−6.940**
					(2.431)
Constant	41.155***	−0.444	−0.897**	−0.089	42.632***
	(7.706)	(0.367)	(0.337)	(0.041)	(7.409)
N	773	773	773	773	773

Significance levels: ^+^ < 0.1; * 0.05; ** 0.01; *** 0.001; models contain all individual-level co-variates (although not shown); clustered standard errors; unstandardized coefficients

Model 1 (Table 5) replicates the positive but non-significant association between workplace exposure and attitudes. Models 2–4 test the effect of workplace diversity on the frequency of contact reported as either high-, medium- or low-enjoyment. As observed in neighbourhoods, workplace exposure is positively associated with *all* types of contact (although these associations are stronger in workplaces).^22^ Thus, in more diverse workplaces we observe a higher number of individuals exhibiting more frequent positive contact (i.e. high and medium enjoyment), but also negative contact (i.e. low enjoyment); although diversity has a stronger association with frequency of positive contact.^23^ These results provide support for H1 for workplaces.

We next explore how ‘low-’, ‘medium-’ and ‘high-enjoyment’ workplace contact are associated with inter-group attitudes (Model 5). Individuals reporting more ‘high-enjoyment’ contact exhibit more positive attitudes; those individuals reporting more ‘low-enjoyment’ contact exhibit more negative attitudes; however, there is no significant effect of ‘medium-enjoyment’ contact on attitudes. These findings provide support for H2 across workplaces. Furthermore, while high-enjoyment contact has similar effects across workplaces and neighbourhoods,^24^ medium-enjoyment has a weaker (and non-significant) association in workplaces,^25^ while low-enjoyment contact has a weaker negative effect across workplaces.^26^

**Fig. 4:**
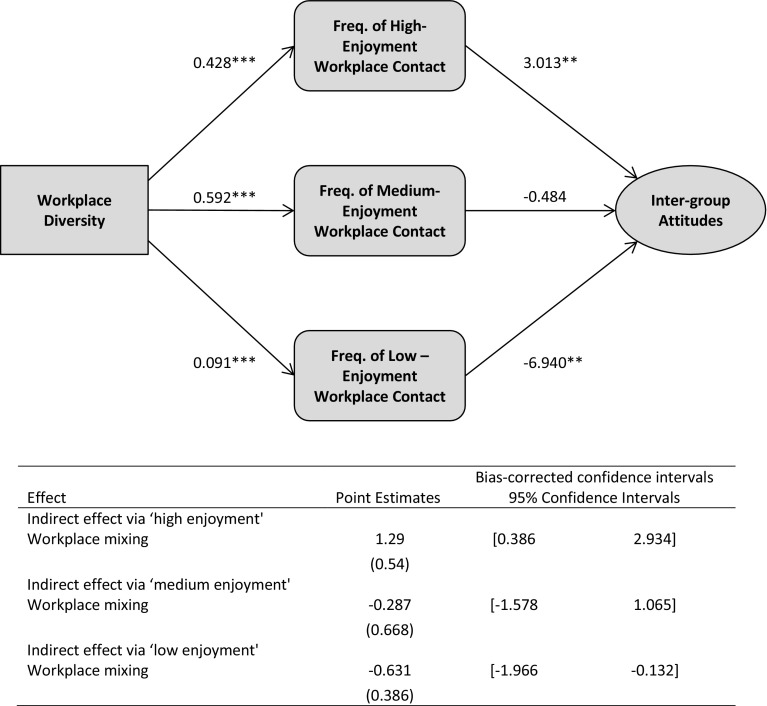
Mediation model showing effect of workplace diversity on out-group attitudes through frequency of ‘low-’, ‘medium-’ and ‘high-enjoyment’ inter-group contact. *Notes*: Unstandardized coefficients are shown, and bootstrap standard errors are given in parentheses; when confidence intervals do not include zero, value of the indirect effect is significant at the *p* ≤ 0.05 level; all results were controlled for individual-level and community-level covariates

We posited that such pathways could drive *both* positive and negative indirect effects of diversity on attitudes via contact. We again model these pathways simultaneously in a structural equation model; Fig. 4 summarises these results. As across neighbourhoods, there is a positive indirect-effect of exposure via those individuals who report more high-enjoyment mixing, and a negative indirect-effect via those individuals who report more low-enjoyment mixing; although there is no indirect-effect via individuals who report more medium-enjoyment mixing.^27^ These findings also substantiate H3 across workplaces. However, substantive differences exist in these processes across workplaces. Workplace exposure has stronger positive indirect-effects via high-enjoyment contact, but also a stronger negative indirect-effect via low-enjoyment contact; it also has a (non-significant) negative indirect-effect via medium-enjoyment mixing (compared to the significant positive indirect-effect across neighbourhoods).^28^

We next want to explore how these countervailing indirect contact-pathways can help understand the overall workplace diversity/prejudice association. Model 1 (Table 6) once again establishes the positive but non-significant association between workplace exposure and out-group warmth. One possibility is that those individuals reporting more negative inter-group mixing at higher workplace exposure may be suppressing an otherwise positive effect of diversity on attitudes. To test this idea we examine the association between workplace exposure and attitudes but adjust for those individuals who report more frequent ‘low-enjoyment’ contact (Model 2): the positive coefficient for workplace exposure becomes stronger and is now significant. After adjusting for those individuals who report more ‘medium-enjoyment’ contact the positive association (and significance) of exposure again increases (Model 3).

**Table 6:** Changing effects of workplace diversity with (valenced) inter-group contact

Model type	Model 1	Model 2	Model 3	Model 4
	OLS	OLS	OLS	OLS
Outcome	Feeling therm.	Feeling therm.	Feeling therm.	Feeling therm.
Workplace diversity	1.598	2.229*	4.102***	1.041
	(1.207)	(1.135)	(1.160)	(1.459)
‘High enjoy’ work mixing				3.197**
				(1.062)
‘Medium enjoy’ work mixing			−2.919***	−0.537
			(0.827)	(1.088)
‘Low enjoy’ work mixing		−7.050**	−8.832***	−6.021*
		(2.579)	(2.627)	(2.758)
_cons	41.155***	41.785***	39.328***	42.632***
	(7.706)	(7.601)	(7.625)	(7.409)
N	773	773	773	773

Significance levels: ^+^ < 0.1; * 0.05; ** 0.01; *** 0.001; models contain all individual-level co-variates (although not shown); clustered standard errors; unstandardized coefficients

Therefore, once we adjust for those individuals who report more ‘low-’ and ‘medium-enjoyment’ mixing at higher exposure, workplace exposure has a positive and significant association with inter-group attitudes. This positive association may be driven by those individuals who report more ‘high-enjoyment’ mixing at higher exposure. Adjusting for rates of ‘high-enjoyment’ mixing, the direct positive exposure coefficient is reduced by 76% and rendered non-significant^29^ (Model 4). These results do not confirm H4a and H4b across workplaces.

### Measurement, Causality and Robustness

**Fig. 5:**
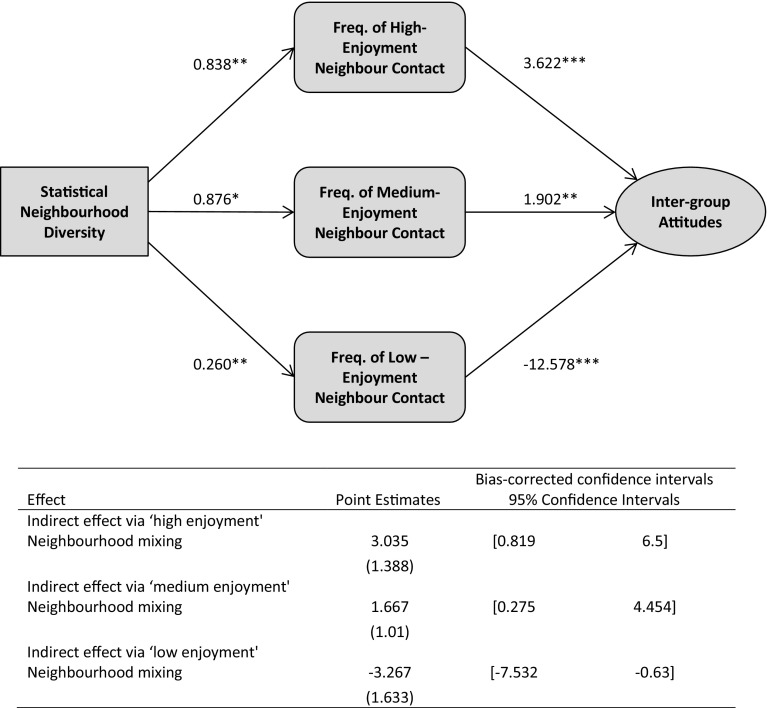
Mediation model showing effects of statistical neighbourhood diversity on out-group attitudes through frequency of ‘low-’, ‘medium-’ and ‘high-enjoyment’ inter-group contact. *Notes*: Unstandardized coefficients are shown, and bootstrap standard errors are given in parentheses; when confidence intervals do not include zero, value of the indirect effect is significant at the *p* ≤ 0.05 level; all results were controlled for individual-level and community-level covariates

We address issues with the preceding analysis. Firstly, we examine how much self-reported exposure measures bias our analysis by re-running our neighbourhood analysis using actual percent non-white British in the community. To test this idea we replicate the SEM results observed for self-reported neighbourhood diversity (Fig. 3) but using actual neighbourhood diversity (Fig. 5).

Comparing the actual/self-reported neighbourhood SEM models, only slight differences emerge, and on the whole, the same substantive conclusions can be drawn. This increases confidence that self-reported neighbourhood (and, to a lesser extent, workplace) exposure is closely related to actual exposure.

Another issue concerns the association between out-group exposure and positive/negative contact. Firstly, respondents in homogeneous sites cannot experience contact. Secondly, only respondents who reported having at least some contact were asked whether it was ‘enjoyable or not’. Therefore, the positive associations between exposure and both positive and negative contact may emerge simply by virtue of the way in which these measures were created: if people in homogeneous areas cannot experience contact, and being asked the valence-question is conditional on having contact, we would expect a positive correlation between exposure and valenced-contact. However, what is important is that higher exposure is not simply associated with experiencing (positive/negative) contact or not; it is associated with a greater *frequency* of positive/negative contact. This is critical, because having more frequent positive/negative contact is associated with better/worse inter-group outcomes than experiencing infrequent positive/negative contact *or* no contact. Therefore, exposure has positive/negative indirect effects on attitudes via contact not simply because individuals in homogeneous areas cannot experience (positive/negative) contact, but also because it is associated with *more frequent* experiences of positive/negative contact.

We further tested this idea by re-running all our analysis but restricting our sample to individuals who experienced at least some site-specific contact. This returned substantively similar findings; therefore, even excluding the initial association between exposure and whether a respondent has contact or not, the key findings still hold (*results available on request*). We also formally tested moderated-mediation models (in which exposure predicts contact-quantity which predicts inter-group attitudes moderated by contact-quality). These analyses similarly demonstrate the importance of the quantity of positive/negative contact for the exposure/inter-group attitudes relationship (see Appendices 1, 2).

Another issue concerns what drives contact-valence. As previously discussed, contact-valence may reflect the nature of the interaction itself (e.g. being helped/harmed) and/or be driven by individual-predispositions. To gain some purchase on this issue in our study we examine the association between how much individuals ‘enjoy the mixing occurring in their workplace’ and ‘enjoy the mixing occurring in their neighbourhood’. If ‘enjoyableness of contact’ is largely driven by some latent disposition then we would expect an individual’s reported enjoyableness of workplace and neighbourhood contact to be highly correlated (as any differences in the nature of the interactions in workplaces and neighbourhoods themselves would matter less). Enjoyableness of workplace mixing is correlated with enjoyableness of neighbourhood mixing at: *r* = 0.48, suggesting there may be a degree of latent propensity. However, the correlation is only moderately strong, suggesting a latent disposition towards experiencing *any* contact positively/negatively is unlikely to account for all the valence of our contact measures.^30^ Yet, even if ‘enjoyableness’ is picking up some latent predisposition, the findings still suggest that experiences of contact matter for respondents’ inter-group attitudes as a result of the *amount of contact* they experience. For example, those individuals who report negative contact may be more predisposed to experience any contact they have more negatively. However, what we see is that those who experience *more frequent* negative mixing at higher exposure are likely to report greater prejudice than those with equally negative predispositions who experience *less frequent* mixing^31^ (i.e. that more frequent contact itself could still drive greater prejudice if it occurs amongst those predisposed not to enjoy it).

 Lastly, drawing on the current exposure-contact-prejudice causal framework, we suggested that exposure leads to greater amounts of positive/negative contact, which leads to lower/higher prejudice. However, our data are cross-sectional and some pathways could operate in other directions. One key issue is that studies show that prior levels of prejudice can affect one’s likelihood of engaging in contact (Binder et al. 2009), while one’s attitudes towards out-groups can also influence whether the inter-group contact one experiences is perceived as more positive/negative (Stephan and Stephan 1985). Our findings demonstrate that individuals who have higher exposure and who report *more frequent* positive (or negative) contact, report lower (or higher) prejudice. One alternative explanation is that low prejudice causes individuals to enjoy mixing with out-groups. This may cause them to choose to mix more frequently, and also potentially select into more diverse neighbourhoods/workplaces. This could account for why some individuals at higher exposure, who report more frequent positive contact, report low prejudice. Equally, it is feasible that higher prejudice causes individuals to not enjoy mixing with out-groups. However, this would be unlikely to cause individuals to choose to interact more frequently, or to select into diverse contexts. Yet, it is those who experience more frequent negative contact (who tend to be found in diverse contexts) who report higher prejudice. Therefore, the negative indirect pathway (exposure-high negative contact-high prejudice) is less susceptible to reverse-causal explanations.

We are unable to test causality further here. However, our findings that individuals reporting *more frequent* unenjoyable-contact report more prejudice, that such individuals are more likely to be found at higher exposure, and that this association accounts for part of the exposure-prejudice association, remain compelling.

## Discussion and Conclusion

Prior studies into the effect of out-group exposure on inter-group attitudes suggest diversity generates greater inter-group contact and that this greater mixing will either drive a positive overall effect of diversity (ecological-contact hypothesis) or counteract an otherwise negative effect of diversity that is driven by perceived-threat. We argued that this theoretical framework may only partially explain how exposure affects attitudes via contact as it overlooks contact-valence. We integrated the concept of contact-valence into the current exposure/contact/prejudice framework, positing that exposure may affect attitudes through both positive- *and* negative-contact pathways. We demonstrate evidence in support of this model.

We first replicated the approach of much of the current literature. Using a measure of generic inter-group mixing, we observe a positive indirect-effect of (neighbourhood/workplace) exposure on inter-group attitudes via individuals reporting more frequent generic-contact. In the case of neighbourhoods, this pathway suppresses an otherwise significant negative direct association between exposure and attitudes. Across workplaces, this indirect-effect drives almost all of the positive (albeit non-significant) association between exposure and attitudes.

Integrating contact-valence into this analysis highlights the limits of this approach. At higher (workplace/neighbourhood) exposure we observe a greater number of individuals who report more positively-valenced mixing, and this positive mixing is associated with lower prejudice. However, exposure is also associated with a greater number of individuals who report more negatively-valenced mixing, who in turn report higher prejudice. Exposure therefore has *both* positive and negative indirect-effects on inter-group attitudes *operating through contact pathways*.

Accounting for these positive/negative pathways also changes our understanding of what drives the overall association between exposure and prejudice. In neighbourhoods, the negative effect of exposure on out-group attitudes is only suppressed by those individuals who report more positively-valenced contact in diverse communities (and not all forms of contact). Once these positive-contact pathways are accounted for, neighbourhood exposure has a negative association with attitudes. However, a substantial part (and the statistical significance) of this negative association can be accounted for by the presence of individuals who report more frequent negative-contact at higher exposure. Across workplaces, more frequent negatively-valenced contact in diverse workplaces suppresses an otherwise significant positive effect of workplace exposure on attitudes. This positive association is largely driven by the presence of individuals who report frequent positively-valenced mixing at higher exposure.

In sum, inter-group contact can drive a positive effect/suppress a negative effect of contextual ethnic diversity as prior studies show; however, this only occurs through more positively-valenced contact. At the same time, more negatively-valenced contact can drive a negative effect/suppress a positive effect of diversity. Therefore, underlying the overall effect of exposure on inter-group attitudes are dual, countervailing pathways of positive and negative inter-group contact.

Evidence of these processes across both workplaces and neighbourhoods demonstrates the generalisability of this modified ecological-contact hypothesis. Yet, differences between contexts exist. All contact increases at a greater rate with workplace exposure compared to neighbourhoods. Workplaces may encourage greater out-group mixing through the necessity of mixing with colleagues more generally for work purposes (and thus out-groups by association). In neighbourhoods, there are fewer obligations to mix generally, while individuals can also actively choose not to mix. The necessity to interact in workplaces may also restrict homophilic tendencies, which can be exercised more in neighbourhoods.

Another difference between contexts is the effect size of valenced-contact. Across both contexts, increasing ‘low-enjoyment’ mixing has a stronger effect than increasing ‘high-enjoyment’ mixing (Barlow et al. 2012). However, while ‘high-enjoyment’ contact has broadly similar effects across contexts, the negative effect of ‘low-enjoyment’ contact is almost twice as strong in neighbourhoods. Potentially, workplace environments could limit the strength of negative contact experiences. Workplaces necessitate repeated interactions over time. This may enforce a level of civility and accountability on interactions, which are not present in neighbourhoods where actors can choose whether to interact again. Furthermore, formalised codes of behaviour/conduct (such as anti-bullying policies), attendant mediation structures, alongside informal social norms of behaviour may also limit the occurrence of severe negative experiences in workplaces. The lack of such constraints on behaviour in neighbourhoods may provide greater scope for stronger negative experiences. Another possibility is that perceived-threat may be more likely to emerge from neighbourhood (rather than workplace) exposure. The presence of threat may lead to stronger negative contact experiences for some in neighbourhoods (Pettigrew and Tropp 2006). However, at the same time, higher medium-enjoyableness contact is not positively associated with attitudes in workplaces. This could emerge from the greater scope for less intimate but more instrumental, unvalenced contact in workplaces.

In spite of these differences, on the whole, contact appears to account for a similar amount of the overall effect of neighbourhood/workplace exposure on attitudes.^32^ The net-effect of all valenced-contact pathways is positive: in workplaces, adjusting for all valenced-contact measures reduces the positive direct association of diversity by 35%; in neighbourhoods, it increases the negative direct association of diversity by 33%. This similar positive net-effect occurs for a combination of reasons. In workplaces, although exposure is associated with greater mixing than in neighbourhoods this increases the amount of both positive and negative mixing, operating to cancel one another out. Furthermore, although the indirect-effect of high-enjoyable contact is much stronger across workplaces than neighbourhoods, there is no indirect-effect via medium-enjoyableness contact in workplaces. In neighbourhoods, the positive indirect-effects of both high- *and* medium-enjoyableness contact are suppressed by the much stronger negative effects of low-enjoyableness contact.

Processes operating outside of inter-group contact, however, also likely contribute to context-specific differences in the overall effect of diversity on prejudice. In workplaces, after adjusting for all valenced-contact pathways, a positive (albeit reduced, non-significant) association still remains. In comparison, across neighbourhoods, after adjusting for all valenced-contact pathways, a negative (albeit increased, non-significant) association still remains. Furthermore, across workplaces, we see that only controlling for rates of negative contact results in a *significant* (positive) direct effect of exposure; just controlling for rates of positive contact does not result in a significant, negative direct effect of exposure (*results available upon request*). Across neighbourhoods, we observe that only controlling for positively-valenced contact results in a *significant* (negative) direct effect of exposure; however, just controlling for rates of negative contact does not result in a significant, positive direct effect of exposure (*results available upon request*). Other pathways are thus likely driving part of the overall positive/negative relationships between workplace/neighbourhood exposure and prejudice.

We acknowledge that, notwithstanding the new insights gleaned, this study has some limitations. Firstly, we are unable to examine the role of other workplace socio-demographics in this relationship and how far they may be driving any apparent workplace effects (for example, workplace educational composition), or whether differences in workplace effects exist across different occupations/workplace-types. Thus, we caution against generalising to all workplaces. Secondly, as noted, our data are also cross-sectional, ruling out causal inferences. Thirdly, it focuses on the majority, White British population alone. How these relationships operate amongst minority groups requires further investigation. Lastly, based on the available data, this paper took an ‘overall-valence’ approach to studying positive/negative contact pathways which exert positive/negative indirect-effects through *different* sets of individuals. Future research would benefit from applying measures which also allow individuals to report separate experiences of both positive and negative contact (alongside overall-valence), to test whether positive/negative indirect effects may also operate through the *same* individual.

This study has implications for the application of the contact-hypothesis in contextual-effects studies. While previous studies demonstrate positive-indirect effects of contact, we show that diversity has *both* positive *and* negative *indirect*-*effects* on intergroup attitudes via contact pathways. The omission of this negative-pathway from the original ecological-contact hypothesis may account for the lack of supporting evidence. To be sure, for individuals as a whole the evidence is supportive of the idea that micro-level contact can ameliorate macro-level conflict: importantly, the net-effect of diversity on attitudes via *mixing* is positive. However, as exposure increases, a minority of individuals can become even more hostile towards out-groups as a result *of*, and not due to a lack of, contact. Compared to individuals in low-exposure environments, those individuals experiencing high-exposure who report more frequent negative-contact have worse out-group attitudes relative to those who have less frequent negative-contact or no contact at all. Therefore, diversity appears to also exert a *polarising* effect on attitudes towards out-groups via increasing rates of contact, driving a minority of individuals towards greater hostility.

This study also has implications for the debate on how exposure affects attitudes more generally. Macro-level studies have previously demonstrated how increasing diversity can lead to higher incidences of both positive and negative contact (Blau and Schwartz 1984). This study reconfirms this idea, and shows how such contextual-diversity translates into experiences of both positive and negative contact amongst different individuals. However, it also demonstrates the consequences of such greater positive and negative contact, helping further elucidate the mechanisms by which exposure affects inter-group relations more generally. Previously, after accounting for (positive/generic) inter-group contact, studies largely attribute any negative effects of exposure to a psychological pathway of perceived-threat. Problems are thus perceived to stem from a lack of mixing. However, greater inter-group mixing itself can have a negative-effect via more negatively-valenced contact. This is not to say that perceived threat plays no role. It may account for the remaining negative effect of exposure (operating alongside negative-contact), or be the reason why some individuals experience encounters negatively in more diverse environments. Moreover, it may be that perceived-threat is driven, in part, by negative encounters. Prior research has found that positive contact reduces threat; it is thus likely that negative contact in high exposure contexts increases threat, which then further drives the negative effects on prejudice.

This reasoning suggests that the oft-advocated aim of greater inter-group mixing may not alone produce the intended outcomes of prejudice reduction with increasing diversity. Although we do observe that the net-effect of exposure via contact is positive, we also stress that more careful attention needs to be paid to contact-valence, and the *conditions* that produce more positive/less negative contact; especially given these contact pathways appear to polarise attitudes as diversity increases. As demonstrated, these processes appear to operate differently across neighbourhood- and workplace-exposure. While we discuss some possible reasons for these site-specific effects, future research will benefit from examining what macro-level *and* micro-level conditions lead to more positive and/or negative contact.

Finally, this study also highlights limitations with the existing evidence-base used to claim that diversity poses a threat to inter-group relations; in particular, its narrow focus on residential communities. We undertook the first joint-comparative analysis of neighbourhood and workplace exposure in England. In the realm of neighbourhoods our findings across neighbourhoods appear to substantiate some of the more pessimistic claims regarding diversity (although our findings do also offer support for the more optimistic prediction that micro-level positive contact can suppress prejudicial attitudes with increasing neighbourhood diversity). In the workplace realm, however, exposure generates comparatively *more* positive inter-group attitudes in our sample, which has important implications for strategies to tackle issues of integration. Specifically, more focus should be given to the workplace as a possible context for integration. Policies that limit employment discrimination, which encourage diverse workforces, as well as efforts to eliminate ethnic *and* socio-economic inequalities in access to, and participation in, all levels of the workforce, could play a key role in fostering positive inter-group relations more generally.

## Appendix 1

See Table 7.

**Table 7:** Moderated-mediation model of the association between exposure, contact-quantity and -quality, and inter-group attitudes across neighbourhoods

Model type	M1	
	SEM (OLS)	
Outcome	Quantity of neighbourhood mixing	Feeling thermometer
Neighbourhood diversity	0.316***	−1.863^+^
	(0.055)	(0.953)
Quantity of neighbourhood mixing		−2.712
		(1.905)
Enjoyment of neighbourhood mixing		−0.131
		(1.388)
Quantity × enjoyment		1.412*
		(0.595)
Constant	1.917***	47.251***
	(0.442)	(8.923)

Effect	Point estimates	Bias-corrected confidence intervals 95% confidence intervals	
Indirect effect: *minimum* contact-quality	−0.994	[−2.054	0.066]
	(0.541)		
Indirect effect via *maximum* contact-quality	0.894	[0.206	1.782]
	(0.402)		

Significance levels: ^+^ < 0.1; * 0.05; ** 0.01; * 0.05; **  0.01; *** 0.001; models contain all individual-level and community-level co-variates (although not shown); clustered standard error; unstandardized coefficients; *, **, ** used within the tables themselves to signify different levels of statistical significance of the coefficients

Unstandardized coefficients are shown, and bootstrap standard errors are given in parentheses; When confidence intervals do not include zero, value of the indirect effect is significant at the *p* ≤ 0.05 level; all results were controlled for individual-level and community-level covariates

## Appendix 2

See Table 8.

**Table 8:** Moderated-mediation model of the association between exposure, contact-quantity and -quality, and inter-group attitudes across workplaces

Model type	M1	
	SEM(OLS)	
Outcome	Quantity of workplace mixing	Feeling thermometer
Workplace diversity	0.815***	0.238
	(0.040)	(1.426)
Quantity of workplace mixing		−8.695***
		(2.555)
Enjoyment of workplace mixing		−3.925
		(3.856)
Quantity × enjoyment		3.513**
		(1.132)
Constant	1.079***	61.183***
	(0.221)	(8.744)

Effect	Point estimates	Bias-corrected confidence intervals 95% Confidence Intervals	
Indirect effect: *minimum* contact-quality	−7.086	[−11.421	−2.752]
	(2.212)		
Indirect effect via *maximum* contact-quality	4.367	[−0.045	8.779]
	(2.251)		

Significance levels: ^+^ < 0.1; * 0.05; ** 0.01; *** 0.001; models contain all individual-level co-variates (although not shown); clustered standard error; unstandardized coefficients; *, **, ** used within the tables themselves to signify different levels of statistical significance of the coefficients

Unstandardized coefficients are shown, and bootstrap standard errors are given in parentheses; When confidence intervals do not include zero, value of the indirect effect is significant at the *p* ≤ 0.05 level; all results were controlled for individual-level and community-level covariates

## Footnotes

1 Exposure refers to a state of being proximal in space to out-groups, operating at a contextual-level. Crucially, while it increases opportunities for contact it does not *imply* contact is occurring.

2 Weighted self-report measures of workplace ethnic composition for England derived from the 2010 *Managing Cultural Diversity* (MCDS) data, which is the data set used in this paper.

3 By ‘overall effect’ we mean there should be a significant association between our key independent variable (out-group size) and our outcome (inter-group relations) before the inclusion of posited mediation variables.

4 For example, in suggestions that there is little evidence for the ‘contact hypothesis’ when referring to the contact hypothesis as applied to the contextual-level (e.g. Putnam 2007).

5 We use the term ‘suppressing’ here to signify a situation in which the inclusion of a variable in a model (in this case, the mediator) can increase the size and significance of another variable (in this case, the independent variable), i.e., where a mediator suppresses the effect of the independent variable (MacKinnon et al. 2000).

6 For full details see the 2010 Managing Cultural Diversity Survey *Technical Appendix.*

7 Applying self-reported measures as proxies for actual out-group size raises several issues. There are problems of how accurate these measures are in capturing actual out-group size. Some argue that self-reported measures may be superior to statistical measures, given it is perceptions themselves (although, in part, influenced by actual size) that drive prejudice (Alba et al. 2005). Yet, such measures may also be driven by prior-attitudes towards out-groups e.g., those with higher prejudice over-estimating out-group size, leading to endogeneity between exposure and prejudice.

8 Spontaneous responses of ‘no-one with this background lives in the neighbourhood’ were coded as ‘never’ for this variable.

9 Using such an interaction we could then apply moderated-mediation models to test for indirect-effects of exposure on prejudice via positive/negative contact. Yet, the disadvantages of this approach outlined (such as the loss of the ‘no contact’ individuals) reduce the effectiveness of this method. Thus we choose an alternate method of testing our hypotheses. However, to test the sensitivity of our analyses to different specifications we do replicate our analysis using a moderated-mediation approach (see Appendices 1, 2).

10 How other studies in the literature deal with this issue without excluding those ‘no contact’ individuals is unclear.

11 ‘Enjoy quite a bit’ was classed as medium-enjoyableness to create a more even *n* in each category. However, coding it as high-enjoyment contact did not result in substantial differences.

12 This is considered a more robust method of calculating indirect effects as it does not require a normal sampling distribution.

13 We also ran the workplace and neighbourhood analysis in a single model to test whether effects at one site were being driven by effects across the other. However, the same substantive conclusions could be drawn. For ease of interpretability as well as minimising potential issues of multicollinearity we report our workplace/neighbourhood analysis from separate models.

14 We replicated all models excluding non-workers. This did not change our substantive conclusions.

15 If the confidence intervals do not include zero the value of the indirect effect is taken as significant at the *p* ≤ 0.05 level.

16 The difference between the association of diversity with high-enjoyment and low-enjoyment contact is significant at *p* value <0.1.

17 The coefficients for exposure on ‘low-’, ‘medium-’ and ‘high-enjoyment’ contact sum to the coefficient for exposure on generic contact.

18 We also applied a moderated-mediation analysis to test for robustness across different specifications. Although these models exclude individuals experiencing ‘no contact’ (who have no contact-quality score) and assume an equal opportunity of experiencing high-/low-quality contact, Appendix 1 shows substantively similar findings of positive/negative indirect-effects.

19 Difference with neighbourhood exposure significant at *p* value <0.05.

20 Significant at *p* value <0.001.

21 The significance of workplace mixing is at *p* value <0.1. However, this is driven by a degree of collinearity with workplace exposure (*r* = 0.69). The workplace mixing coefficient exhibits a similar size but greater significance if we exclude workplace diversity from the model (*b* = 2.14**).

22 The associations with medium- and high-enjoyment contact are significantly stronger (to a *p* value <0.001 level). The association with low-enjoyment contact is stronger but not significantly so.

23 Workplace exposure has a significantly stronger positive association with medium- and high-enjoyment contact than low-enjoyment contact (at a *p* value <0.001 level).

24 No significant difference in coefficient sizes.

25 This difference with neighbourhoods is significant at a *p* value <0.05 level.

26 This difference with neighbourhoods is significant at a *p* value <0.001 level.

27 Appendix 2 shows the results of the moderated-mediation approach to this analysis, again demonstrating substantively similar findings.

28 However, comparing indirect-effects confidence intervals across SEM models (Figs. 3, 4) demonstrates that these substantive differences are not significant.

29 The significant negative coefficient of ‘medium-enjoyment’ mixing in Model 3 (compared to Model 4) emerges because this association is compared to individuals who experienced ‘high-enjoyment contact’ (as this latter group are not included/controlled for in Model 3 as they are in Model 4).

30 The observed model relationships also hold if we include in the models proxies for inter-group threat (e.g. ‘People with an ethnic minority background take good jobs away from White British people’). This suggests both contact-valence and inter-group attitudes are not being driven by some element of shared out-group threat.

31 This is also demonstrated in the formalised moderated-mediation models (see Appendices 1, 2).

32 The total indirect-effect of exposure via all contact pathways is *b* = 0.37 for workplaces and *b* = 0.42 for neighbourhoods.
